# Editorial: Unravelling microbial interactions in plant health and disease dynamics

**DOI:** 10.3389/fmicb.2025.1716380

**Published:** 2025-11-17

**Authors:** Abhijeet Shankar Kashyap, Nazia Manzar, Parul Chaudhary, Sajad Ali

**Affiliations:** 1 Plant Pathology Lab, Indian Council of Agricultural Research (ICAR)-National Bureau of Agriculturally Important Microorganism, Mau, India; 2 Department of microbiology, Graphic Era Hill University, Dehradun, India; 3 Department of Biotechnology, College of Life and Applied Sciences, Yeungnam University, Gyeongsan, Republic of Korea

**Keywords:** microbial interactions, plant health, disease dynamics, pathosystems, microbiome

## Introduction

Plants exist in a dynamic microbial environment where symbionts, pathogens, and commensals continuously interact, shaping plant growth, immunity, and disease outcomes. These interactions occur across scales from molecular recognition at the cell wall to ecosystem level microbiome shifts and profoundly influence food security, crop productivity, and sustainable agriculture. This Research Topic, *Unravelling microbial interactions in plant health and disease dynamics*, brought together original research and reviews that span plant–microbe symbioses, pathogen genomics, microbiome engineering, and integrative disease management strategies. Collectively, the 21 contributions highlight the dual role of microbes as both allies and adversaries, revealing new molecular insights, diagnostic advances, and innovative biocontrol solutions.

## Symbiotic and beneficial associations: harnessing the microbial wealth

Several studies focused on beneficial microbes and their capacity to support plant health. *Streptomyces coelicolor* was shown to colonize wheat roots endophytically and promote in planta production of ergothioneine, a nutraceutical amino acid with human health implications, offering a striking “soil-to-human health” link (Pipinos et al.). Similarly, grapevine endophytes and chickpea-associated *Trichoderma* isolates demonstrated strong antagonistic activity against fungal pathogens, with biochar and volatile organic compounds (VOCs) enhancing biocontrol efficacy (Holkar et al.; Kumari et al.). In tomato, synergistic amendments of humic acid, chitosan, and *Bacillus subtilis* reshaped the rhizosphere microbiome and reduced disease burden, underscoring the importance of microbial consortia and bioactive amendments for integrated crop health management (Qiu et al.).

## Microbiome shifts and biocontrol in complex pathosystems

Microbiome reconfigurations in diseased systems were prominently featured. Fusarium-induced avocado root rot disrupted beneficial bacterial taxa, but targeted inoculation with *Bacillus siamensis* restored microbial balance and suppressed the pathogen (Wang et al.). In pine wilt disease, *Pinus koraiensis* endophytes shifted markedly under nematode infection, with fungal dominance increasing as bacterial diversity declined (Li et al.). Such findings emphasize that pathogen invasion is as much a disease of the microbiome as of the host, highlighting opportunities for microbiome-guided biocontrol interventions.

## Genomics and molecular dissection of pathogen virulence

A significant theme was pathogenomics and functional gene analysis. Genomic and secretome profiling of *Rhizoctonia solani* from proso millet revealed virulence factors, CAZymes, and adaptive evolution (Koti et al.). Similar genomic-scale approaches unraveled roles of GH3 hydrolases in *Fusarium verticillioides* (Zhang et al.), GH11 xylanases in *Neostagonosporella sichuanensis* (Liu, Li et al.), and SDR genes in *Arthrinium phaeospermum*, all contributing to virulence and host penetration (Liao et al.). Mixed-strain infections of *Magnaporthiopsis maydis* highlighted the complexity of within-species variation and its impact on resistance durability in maize (Shofman et al.). Meanwhile, the myxobacterium *Cystobacter fuscus* emerged as a novel predator of *Verticillium dahliae*, secreting hydrolytic enzymes with potential for biocontrol (Han et al.). These contributions advance our mechanistic understanding of microbial virulence and pave the way for functional genomics-driven control strategies.

## Viral threats and vector–microbe interactions

Plant viral pathosystems were also well-represented. Screening of common bean genotypes from the North-Western Himalayas identified key resistance genes against BCMV and BCMNV, offering valuable resources for breeding (Meghanath et al.). In temperate pome fruits, a review synthesized the diverse viral threats and their management. Metagenomic profiling of whiteflies transmitting Tomato Leaf Curl Virus (ToLCuV) in India revealed the critical role of endosymbiotic bacterial communities in shaping vector competence (Manzoor et al.). Integrative systems biology further demonstrated how chloroplast- and mitochondria-associated genes regulate host defense during viral replication. Together, these studies highlight the intricate triangular interactions among virus, vector, and host (Shahriari et al.).

## Host defense modulation and co-infection dynamics

Two contributions focused on how pathogens manipulate host immunity during complex infections. Co-infection of tobacco by *Ralstonia solanacearum* and *Phytophthora parasitica* amplified disease severity by deregulating ROS metabolism and downregulating PR genes, providing a framework for understanding multi-pathogen interactions (Liu, Wang et al.). In rubber tree, a cerato-platanin protein from *Rigidoporus microporus* triggered ROS accumulation, callose deposition, and defense gene activation, expanding the functional spectrum of elicitors in perennial crops (Maiden et al.). Additionally, studies on *Pseudomonas syringae pv. tomato* infection showed how bacterial disease disrupts the entry of non-pathogenic immigrants into the apoplast, emphasizing how infections reshape microbial niches within the host (Cowles et al.).

## Durable resistance and epidemiological insights

Wheat leaf rust resistance was dissected across Indian genotypes, revealing both seedling resistance genes (Lr1, Lr10, Lr24, etc.) and adult plant resistance mediated by slow-rusting minor genes (Mohan et al.). The combination of molecular markers and epidemiological parameters underscores the utility of multigenic resistance deployment against rapidly evolving pathogens. Similarly, seasonal influences on whitefly dynamics provided epidemiological insights into how climate, cropping systems, and microbial associations govern disease outbreaks.

## Conclusion: toward an integrated understanding of microbial dynamics

This Research Topic collectively illuminates the multi-dimensional roles of microbes in plant health and disease from beneficial associations that boost immunity and growth, to pathogens evolving sophisticated mechanisms of virulence, to entire microbiomes reshaped under disease pressure (Sujatha et al.). Advances in omics, metagenomics, and systems biology are revealing unprecedented complexity, but they also offer new tools for diagnosis, breeding, and biocontrol. The studies presented here reinforce the need for interdisciplinary approaches that integrate microbiome ecology, functional genomics, and applied plant pathology. Looking forward, the challenge lies in translating these mechanistic insights into scalable, field-ready solutions microbial consortia, bioformulations, resistant varieties, and predictive models that can safeguard global food systems under the dual pressures of climate change and pathogen evolution. Revealing the microbial-plant interactions, we move closer to resilient and sustainable agriculture.

